# Stage 1 Registered Report: Variation in neurodevelopmental outcomes in children with sex chromosome trisomies: protocol for a test of the double hit hypothesis

**DOI:** 10.12688/wellcomeopenres.13828.2

**Published:** 2018-04-24

**Authors:** Dianne F. Newbury, Nuala H. Simpson, Paul A. Thompson, Dorothy V. M. Bishop

**Affiliations:** 1 Department of Biological and Medical Sciences, Oxford Brookes University, Oxford, Oxfordshire, OX3 0BP, UK; 2 Department of Experimental Psychology, University of Oxford, Oxford, Oxfordshire, OX1 3UD, UK

**Keywords:** Language disorder, sex chromosome trisomy, autism, genetics, neuroligin, neurexin

## Abstract

**Background**: The presence of an extra sex chromosome is associated with an increased rate of neurodevelopmental difficulties involving language. Group averages, however, obscure a wide range of outcomes.

**Hypothesis:** The 'double hit' hypothesis proposes that the adverse impact of the extra sex chromosome is amplified when genes that are expressed from the sex chromosomes interact with autosomal variants that usually have only mild effects. *Neuroligin-4* genes are expressed from X and Y chromosomes; they play an important role in synaptic development and have been implicated in neurodevelopment. We predict that the impact of an additional sex chromosome on neurodevelopment will be correlated with common autosomal variants involved in related synaptic functions.

We describe here an analysis plan for testing this hypothesis using existing data. The analysis of genotype-phenotype associations will be conducted after this plan is published and peer-reviewed

**Methods:** Neurodevelopmental data and DNA are available for 130 children with sex chromosome trisomies (SCTs: 42 girls with trisomy X, 43 boys with Klinefelter syndrome, and 45 boys with XYY). Children from a twin study using the same phenotype measures will form two comparison groups (Ns = 184 and 186). Three indicators of a neurodevelopment disorder phenotype will be used: (i) Standard score on a test of nonword repetition; (ii). A language factor score derived from a test battery; (iii) A general scale of neurodevelopmental challenges based on all available information. Autosomal genes were identified by literature search on the basis of prior association with (a) speech/language/reading phenotypes and (b) synaptic function. Preselected regions of two genes scoring high on both criteria, *CNTNAP2* and *NRXN1*, will be tested for association with neurodevelopmental outcomes using Generalised Structural Component Analysis. We predict the association with one or both genes will be detectable in children with SCTs and stronger than in the comparison samples.

## Introduction

Developmental language disorder (DLD), a condition in which there are unexplained and persistent difficulties with language acquisition, affects around 7% of children ( Norbury *et al*., 2016). Family studies show that DLD runs in families ( Bishop, 2008), yet it has proved hard to identify any genetic or environmental factors that substantially increase risk. One reason is that DLD appears to be a complex multifactorial disorder where influences of individual genetic variants (alleles) are typically of small effect, and may interact with other genetic factors and with the environment. Indeed, the ways in which disorders pattern in families suggest that common genetic variants that confer risk of language disorder may lead to an autistic phenotype when they occur with other genetic risk factors ( Bishop, 2010). Thus the specific phenotype can depend on the constellation of genetic variants, rather than there being separate risk factors for DLD and autism spectrum disorder (ASD).

Rather than recruiting increasingly large numbers to try to find reliable associations between language disorders and genetic variants in genome-wide studies, one way forward is to study rare disorders that have a large impact on the phenotype, which may point to functional pathways involved in more common forms of disorder. One instance of a striking association between a genetic condition and language disorder in children of normal intelligence is provided by the sex chromosome trisomies (SCTs), each of which affects 1–1.5 per 1000 children ( Nielsen & Wohlert, 1991). In the 1960s, research was initiated to investigate neurodevelopmental outcomes of children with SCT detected on neonatal screening. A systematic review of these studies showed that in all three trisomies there were high rates of speech and language impairment, motor problems, and educational difficulties, despite IQ being within normal limits in most cases ( Leggett *et al*., 2010). Furthermore, studies of samples who have developmental language disorder of unknown cause find an increased prevalence of sex chromosome trisomies ( Simpson *et al*., 2014).

In a study of children with sex chromosome trisomies identified on prenatal screening, Bishop *et al*. (2011) found that 7 of 30 (24%) girls with karyotype 47,XXX, 9 of 19 (47%) boys with 47, XXY and 15 of 21 (71%) boys with 47,XYY had a history of speech and language-therapy, compared with rates of 4% in sisters and 18% in brothers. Furthermore, this same study found that 2 of 19 (11%) boys with 47,XXY, and 4 of 21 (20%) boys with 47,XYY had received a diagnosis of ASD, compared with an estimated national prevalence rate of 0.2% in girls and 0.6% in boys. In addition, many children with SCTs who were not diagnosed with ASD had evidence of communication difficulties on parental report, including pragmatic (autistic-like) problems, in all three karyotypes. More recent research has provided further evidence of a link with autism as well as other neurodevelopmental disorders in boys with a sex chromosome trisomy ( Ross *et al*., 2012).

The impact of a trisomy is influenced by distinctive characteristics of the sex chromosomes. In most cases, the phenotypic effects of SCTs are much less severe than the impact of an autosomal trisomy: Down syndrome (trisomy 21) usually leads to intellectual disability, and most other trisomies are lethal. Viable trisomies usually involve small chromosomes with a low gene count (for example the Y chromosome), where the effects associated with altered gene dosage are less severe. An exception to this rule is the X chromosome. The X chromosome has a relatively high gene count, but the impact of a duplication is relatively mild because mechanisms of inactivation have evolved, such that in typical females, only one copy is active, and in effect, both males and females have one set of functional genes from this chromosome. In trisomies that involve the X chromosome, two copies are inactivated, largely negating the presence of additional genetic material. There are, however, exceptions to this rule, with between 12–20% genes escaping inactivation to some extent: These include genes in the pseudo-autosomal region, and other genes that have homologues on the Y chromosome ( Carrel & Brown, 2017).

The fact that there is an increase in problems affecting speech, language and communication in all three sex chromosome trisomies suggests there is an adverse impact of an additional copy of a gene that is expressed and has homologous forms on the X and Y chromosomes. *Neuroligin-4 (NLGN4)* is a strong candidate for such a gene, for several reasons ( Bishop *et al*., 2011). First, *NLGN4X*, located on Xp22, escapes inactivation ( Berletch *et al*., 2011). Second there is a homologous gene, *NLGN4Y* on the Y chromosome at Yq11.2. Third, *neuroligins* are expressed in brain, as well as other tested tissues ( Abrahams & Geschwind, 2010; Jamain *et al*., 2003). Fourth, as reviewed by Cao & Tabuchi (2017), mutations of *NLGN4* have been linked to ASD ( Jamain *et al*., 2003; Laumonnier *et al*., 2004; Lawson-Yuen *et al*., 2008; Marshall *et al*., 2008; Pampanos *et al*., 2009; Talebizadeh *et al*., 2006; Yan *et al*., 2008) – although this finding is inconsistent and other studies have not found autism in those with mutations of *NLGN4* ( Chocholska *et al*., 2006; Macarov *et al*., 2007), or have failed to find abnormalities of *NLGN4* in those with autism ( Blasi *et al*., 2006; Gauthier *et al*., 2005; Liu *et al*., 2013; Vincent *et al*., 2004; Yanagi *et al*., 2012). Fifth, neuroligins are postsynaptic transmembrane proteins that mediate development of functional synapses between neurons and are in the same functional network as neurexins ( Craig & Kang, 2007), which have also been implicated in both DLD and ASD ( Vernes *et al*., 2008). Jamain *et al*. (2003) proposed that a defect in *NLGN4* may abolish formation or function of synapses involved in communication. Note that these authors also implicated another X-chromosome neuroligin, *NLGN3,* in autism, but this is located at Xq13, where one copy would be inactivated, and there is no homologue on the Y-chromosome. Therefore, unlike *NLGN4*, *NLGN3* would not be over-expressed in those with an extra X or Y chromosome.

For the reasons described above, we may hypothesise that an extra copy of *NLGN4* could be implicated in neurodevelopmental problems. However, we also need to explain within-karyotype variation. Although there is a substantial increase in rates of speech, language and social communication problems in children with SCTs, the additional chromosome does not cause language impairment or ASD in a deterministic fashion. A minority of children have no evidence of developmental difficulties, a minority are severely affected with disabilities extending across many domains, and most have mild to moderate impairments ( Linden & Bender, 2002).

The wide variation in outcomes suggests that the extra gene dosage could act as a multiplier of other risk factors, which interact with the sex chromosome genes in a dosage-dependent manner and so only assume importance in the subset of individuals who have other genetic or environmental risk factors ( Bishop & Scerif, 2011). This explanation is consistent with rodent research comparing the effect of a *NLGN3* mutation between different strains of mouse, suggesting the impact is dependent on the genetic background ( Jaramillo *et al*., 2017). It also is compatible with evidence from studies of mutations in *NLGN4* in humans, which found that the same mutation may be associated with different phenotypes within one family ( Jamain *et al*., 2003; Laumonnier *et al*., 2004; Lawson-Yuen *et al*., 2008; Yan *et al*., 2005). As well as autism, *NLGN4* associations have been described with intellectual disability, language disorder and Tourette syndrome ( Lawson-Yuen *et al*., 2008; Yan *et al*., 2005).

## Hypothesis

Our pre-planned analysis is designed to test the 'double hit' hypothesis

### The 'double hit' hypothesis: Neuroligins act as a multiplier of effects of neurexins

**Figure 1.**
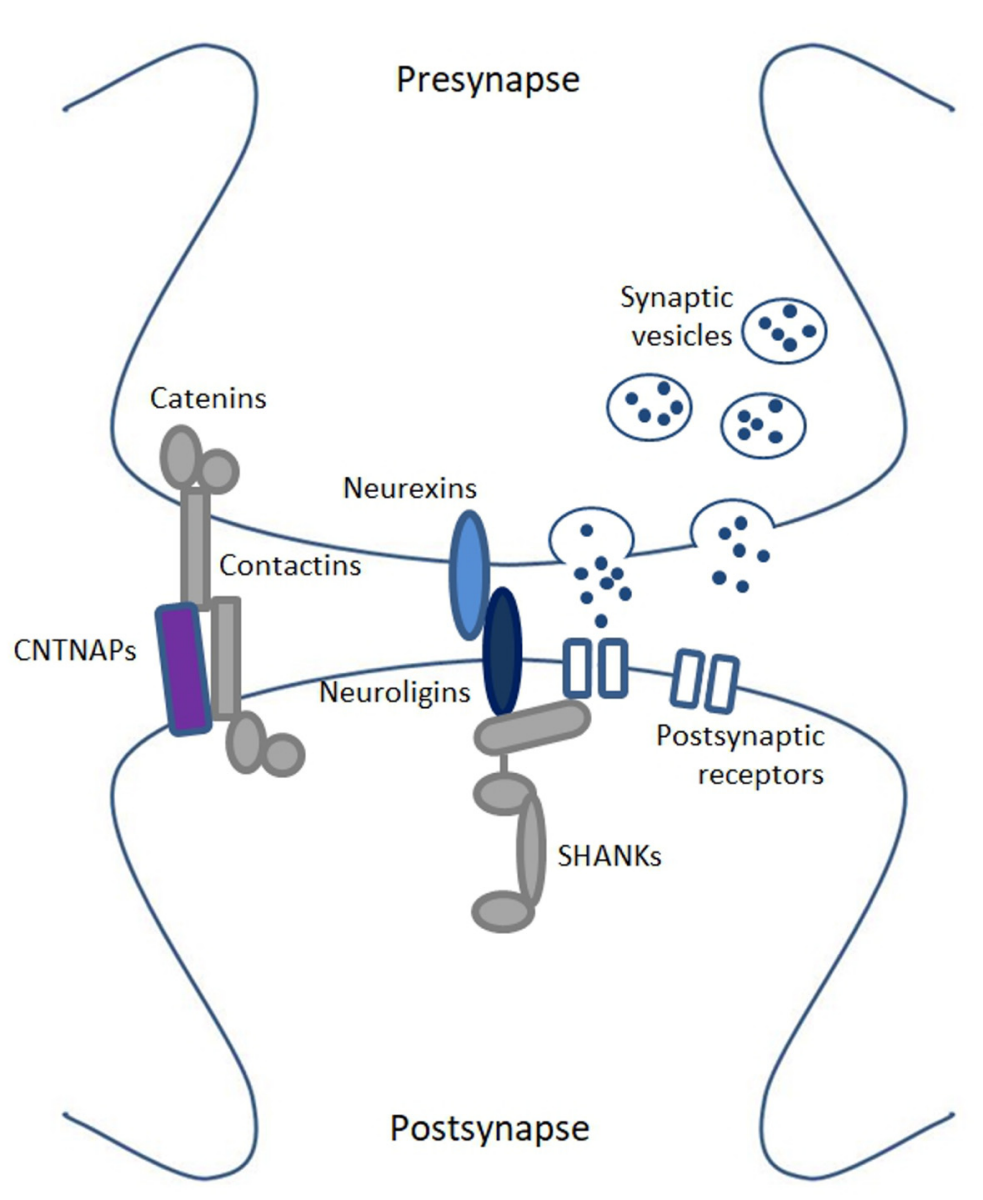
Neurexins (such as *NRXN1*), neuroligins (such as *NLGN4*) and contactin-associated proteins (such as *CNTNAP2*) all form part of the synaptic scaffolding system.

The notion of a ‘double hit’ aetiology has been proposed previously to account for cases where a microdeletion is inconsistently associated with neurodevelopmental disorder ( Girirajan *et al.*, 2010; Newbury *et al.*, 2013): the idea is that a severe phenotype may be seen when there are two copy number variants or mutations, each of which may be relatively innocuous on its own. Here, we extend that idea to argue that the effect of altered *neuroligin* gene dosage may depend on the genetic background provided by autosomes ( Bishop & Scerif, 2011). In this regard, it is of particular interest to note that neuroligin proteins form part of the same functional network as a group of presynaptic transmembrane proteins, known as neurexins; their interactions play a key role in synaptogenesis ( Hussain & Sheng, 2005). *CNTNAP2* encodes a member of the neurexin superfamily whose polymorphisms have been associated with common forms of language impairment ( Graham & Fisher, 2015), though the effect size is relatively small ( Vernes *et al*., 2008). The role of the CNTNAP2 protein in developing brain is not fully understood, and it is likely to play multiple roles at different time-points. While early functional studies of the CNTNAP2 protein indicated that it localises to nodes of Ranvier in axonal membranes, it is now recognised to have key functions at the synapse ( Lu *et al*., 2016; Zweier *et al*., 2009). This raises the possibility that a *CNTNAP2* gene variant that has a modest effect in individuals of normal karyotype might have a much larger impact in the context of overexpression of a *neuroligin*. This hypothesis predicts that presence of an additional sex chromosome will amplify the impact of common genetic variants that have two characteristics: (a) they have been associated with DLD or ASD, and (b) they are in the same functional network as *neuroligins*. Figure 1 shows is a schematic showing two genes of interest to our current study, *CNTNAP*s and Neurexins, interacting with neuroligins in the synaptic cleft.

## Methods

We report how we determined our sample size, all data exclusions, all manipulations, and all measures in the study ( Simmons *et al*., 2012).

### Power analysis and impact of ascertainment bias

We aimed to recruit sufficient children with trisomies to detect an effect size of d = 0.5 for each copy of a given genetic variant on a phenotype, equivalent to a standardized regression slope of 0.25. The anticipated effect size is hard to judge, but the average impact of a sex chromosome trisomy on verbal IQ is more than one SD from the general population mean ( Leggett *et al*., 2010), suggesting that if the trisomy acts as a multiplier of effects of autosomal variants, this effect could be large. When testing variants with a prior association with disorder, we can make a directional prediction. We aimed to recruit 150 children with trisomies, which would have given 94% power to detect a slope of 0.25 on one-tailed test. However, we recruited only 140 children and had missing data on some variables, so numbers, and consequently power, are lower than this. In addition, we have to take into account that the sample is not representative of children with sex chromosome trisomies, because around 50% had the trisomy discovered in childhood when developmental difficulties were being investigated (see below). We devised a simulation to check the impact of these factors on power (see Appendix 5). This showed that a combination of N = 130 with 50% postnatally identified (and presumably biased) cases with mean phenotype score 0.9 SD below the group average (computed from a language factor score), reduced power to 87% on one-tailed test.

### Participants

**Figure 2.**
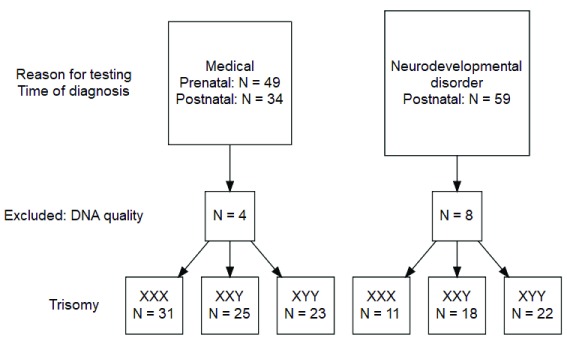
Flowchart showing characteristics of children recruited to SCT group.

Sex chromosome trisomies: After excluding children with missing or inadequate DNA, participants included 42 girls with trisomy X, 43 boys with Klinefelter syndrome, and 45 boys with XYY. These were combined in a single group of 130 children for analysis, but are shown broken down by trisomy and background in Figure 2. Cases were recruited from National Health Service Clinical Genetics Centres, from two support groups ( Unique: the Rare Chromosome Support Group, and the Klinefelter Syndrome Association), or from self-referral via advertisements on the OSCCI website and our Facebook page. A criterion for inclusion was that the child was aware of their trisomy status. In a previous study ( Bishop *et al*., 2011) we noted that levels of impairment tended to be lower in cases where the trisomy was discovered on prenatal screening than in those identified later in childhood. We therefore asked parents specifically about the reason for genetic testing; for 59 children aneuoploidy only came to light because of behavioural or developmental problems. Note that this means that data from this sample should not be used to estimate prevalence of neurodevelopmental disorders in sex chromosome trisomies.

**Figure 3.**
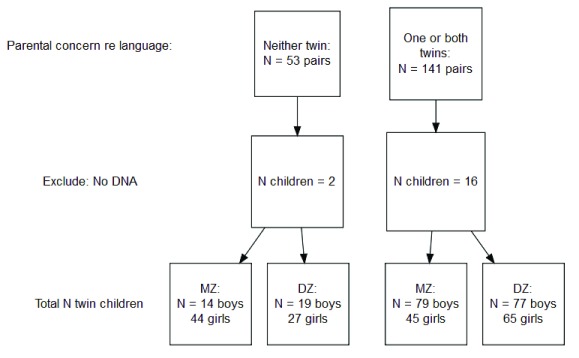
Flowchart showing characteristics of children recruited to comparison groups. Information about zygosity, gender and parental concern is shown for information, but was not used in the analysis. Because twins are not independent, the final sample was divided into two subgroups of 184 and 186 children respectively, each containing one member from each pair, selected at random. (Ns not equal because some twins had missing DNA from just one member of the pair).

Comparison group: Comparison data came from a sample of children aged from 6 years 0 months to 11 years 11 months who had completed the same test battery, who were taking part in a twin study of language and laterality ( Wilson & Bishop, 2018a), and whose first language at home was English. Although twinning is a risk factor for early language delay, this effect appears to wash out with age, and by school age, genetic factors play a major role in the aetiology of language disorder ( Bishop, 2006; Rice *et al*., 2018). In this sample, we aimed for an over-representation of twin pairs in which one or both twins had language or literacy problems that might be indicative of DLD. This was coded on the basis of parental response on a telephone interview: any mention of language delay, history of speech and language therapy, current language problems or dyslexia was coded as 'parental concern'. We aimed to recruit 180 pairs selected on the basis of having language or literacy problems (60 MZ, 60 DZ opposite sex and 60 DZ same sex), and 60 unselected pairs (20 of each type): we fell short of this goal as seen in Figure 3. For the current analysis, we grouped together all twins, regardless of zygosity and parental concern, and then divided them into two subsamples by selecting one twin from each pair at random, after excluding 18 cases with missing or insufficient DNA. This means we can replicate the analysis for twins with a diploid (typical) karyotype. Note that this replication sample is not independent, as the genotype for the MZ twins is the same in the two subsamples, and is related for DZ twins.

Some twin children had evidence of autism spectrum disorder (N = 15) or intellectual disability (N = 3), and twelve failed a hearing screen on the day of testing, although none of them had any known sensorineural hearing loss. For the current study, because we were interested in a broader phenotype than pure DLD, these cases were retained in the sample.

### Test battery

***Psychiatric evaluation.*** In an initial telephone interview, parents were asked about the child's medical and educational history, including a question about whether anyone had diagnosed the child with a neurodevelopmental disorder such as ASD, developmental language disorder (DLD) or specific language impairment, dyslexia or dyspraxia. In addition, one or both parents were asked to complete the online Development and Wellbeing Assessment (DAWBA) ( Goodman *et al*., 2000) in their own time. 84 parents of SCT cases and 133 parents of twins complied with this request. The DAWBA gives information on likelihood of the child meeting criteria for a range of psychiatric diagnoses; a final diagnosis is made by a trained rater who assimilates all the information and evaluates it against DSM5 criteria ( American Psychiatric Association, 2013).

***Language, literacy and cognitive assessments.*** All children were seen at home or in a quiet space in their school for a neurocognitive assessment, using the battery of language and nonverbal ability tests shown in Table 1. Hearing was screened in left and right ears using a DSP Pure Tone Audiometer (Micro Audiometric Corporation). The child was familiarised with the task of raising their hand on hearing a tone using 40 dB (HL) tones. They were then tested with 25 dB pure tones at frequencies of 500, 1000, 2000 and 4000 Hz. Louder tones were presented in 5 dB steps to establish a threshold at any frequency where a 25 dB tone was not detected. Children with an average threshold greater than 30 dB in the better ear were categorized as failing the screen. The battery also included tests of literacy: the Picture and Digit naming tests from the Phonological Assessment Battery ( Frederickson *et al*., 1997), the Test of Word Reading Efficiency ( Torgesen *et al*., 1999) and the Neale Analysis of Reading Ability -2 ( Neale, 1999), but these are not included in the current analysis as there was much missing data from the youngest children. In addition, handedness and language laterality were assessed. Results from laterality assessments were unremarkable and are not considered further here ( Wilson & Bishop, 2018a; Wilson & Bishop, 2018b).

**Table 1.** Assessment battery.

Instrument	Measure
Woodcock Johnson III Tests of Cognitive Abilities ( Woodcock *et al*., 2007)	Verbal Comprehension
NEPSY: A Developmental Neuropsychological Assessment ( Korkman *et al*., 1998)	Repetition of Nonsense Words
	Oromotor Sequences
	Sentence Repetition
Wechsler Abbreviated Scale of Intelligence (WASI) ( Wechsler, 1999)	Vocabulary
	Block Design
	Matrices
Parental questionnaires	
The Children’s Communication Checklist-2 (CCC-2) ( Bishop, 2003)	
The Social Responsiveness Scale (SRS) ( Constantino, 2005)	

For the NEPSY tests, norms extend only to age 12 yr 11 months, and so we used extrapolated scores, as documented in Appendix 1.

### Phenotypes

We will consider three quantitative phenotypes that range from a specific measure of a heritable language skill, through a more general language measure, to a measure that potentially indexes a wide range of neurodevelopmental problems:

A) Nonword repetition, which is regarded as a measure of phonological short-term memory. This was singled out as an individual measure because it has previously been identified in twin studies as a good marker of heritable language problems ( Bishop *et al*., 1996) and has also been associated with genetic variants linked to language/literacy in the *CNTNAP2*, *CMIP*, *ATP2C2*, *KIAA0319*, and *DCDC2* genes ( Carrion-Castillo *et al*., 2017; Marino *et al*., 2012; Newbury *et al*., 2009; Newbury *et al*., 2011; Scerri *et al*., 2011; Vernes *et al*., 2008). In the current study, we used scaled scores from Repetition of Nonsense Words from the NEPSY ( Korkman *et al*., 1998).
B) A general language factor derived from the four other language tests (Verbal Comprehension, Oromotor Sequences, Sentence Repetition and Vocabulary. As documented in Appendix 2, the decision to combined these measures into a single language factor was made after exploring the factor structure of the available phenotypic measures, with the goal of obtaining a reliable indicator of overall language function.
C) A global measure of burden of neurodevelopmental problems extending beyond language, including autistic features. This was developed on an *ad hoc* basis, using all available information from parental report (see Appendix 3).

### DNA collection and analysis

Oragene kits (OG-500, DNA Genotek Inc, Ontario, Canada) were used to collect saliva for DNA analysis from children with SCTs and their parents and available twin pairs. DNA extraction was performed using an ethanol precipitation protocol as detailed in the standard protocol (DNA genotek). All extracted DNA was genotyped on the Infinium ‘ Global Screening Array-24 (v1)’, which includes 692,824 SNPs including rare and common variations. Data were processed in the Illumina BeadStudio/GenomeStudio software (v. 2.03) and all SNPs with a GenTrain (quality) score of < 0.5 were excluded at this stage. All genotypes were further filtered using PLINK software v1.07 ( Purcell *et al*., 2007); as recommended by Anderson *et al*. (2010), samples with a genotype success rate below 95% or a heterozygosity rate ±2 SD from the mean were removed, as were SNPs with a Hardy-Weinberg equilibrium P < 0.000001 or a minor allele frequency of less than 1%. Identity data within families and twin-pairs were used to exclude samples with unexpected gender or relationships. SNPs that showed an inheritance error rate > 1% or skewed missing rates between genotype plates were also excluded. Control data (CEU, YRI, CHB, JPT, Hapmap release #3) were employed through a principal component analysis within Eigenstrat ( Price *et al*., 2006) to identify individuals with divergent ancestry. Sixteen individuals (6 twin pairs and 4 SCT cases) were identified as having African ancestry and 21 individuals (6 twin pairs and nine SCT family members) were identified as having Asian ancestry. Any SNPs that showed a significant association with non-European ancestry (P < 0.0001) were excluded. The final genome-wide dataset consisted of 500 individuals (370 twins, divided into two subgroups, and 130 independent SCT cases) and 451,093 autosomal SNPs with a genotyping rate of 99.78%.

### Procedure

Ethical approval was obtained for the study in 2011 from the Berkshire NHS Research Ethics Committee (reference 11/SC/0096), and data collection started in August of that year, finishing in October 2016. Information sheets, consent forms and ethics approval documents are available on Open Science Framework. Families who had expressed interest in the study were interviewed by telephone to assess whether the child met inclusion criteria, and if so, an appointment was made to see the child at home or at school, depending on parental preference. Families were widely dispersed around the UK, including Northern Ireland, Scotland, Wales and England. During the course of recruitment a total of eight research assistants as well as the senior author were involved in assessing children. The assessment was conducted in a single session lasting between 2–3 hours per child, with breaks where needed.

## Analysis plan

Study data are analysed using R software ( R Core Team, 2016), with the main database managed using REDCap electronic data capture tools hosted at the University of Oxford ( Harris *et al*., 2009).

Potentially, there is a very large number of genotypes and phenotypes that could be analysed to test our hypothesis, as well as different ways of creating subgroups. This consideration, coupled with the small sample size, makes it important to control adequately for multiple testing to guard against type I error ( Grabitz *et al*., 2018). For this reason, we stored phenotype and genotype data separately and specified an analysis plan in detail, as reported here. The analysis of genotype-phenotype associations will be conducted after this plan is registered and peer-reviewed.

### Subgroups

In our main pre-specified analysis we will treat all three trisomies together. This is because the double hit hypothesis postulates a common mechanism that would apply regardless of karyotype. If we find an association between genotype and phenotype, we will carry out exploratory analyses to consider whether this is moderated by karyotype. In particular, we will be in a position to test a prediction by Skuse (2018) that there is more variable expressivity of *NLGN4X* than *NLGN4Y*, which should lead to lower phenotypic variability in XYY compared to the other karyotypes. Note, however, that the ascertainment bias in the sample is problematic for making cross-karyotype comparisons, and the focus would have to be just on those who were not diagnosed because of neurodevelopmental problems (see Figure 2). This is a small sample and so there would be a high risk of missing a true effect (type II error).

### Prioritising genotypes for analysis

We conducted a series of literature searches to prioritise autosomal genes for analysis, focusing on genes that had an association with childhood speech and language disorders and that were relevant for synaptic function (see Appendix 4). This led us to select two candidates; *CNTNAP2* and *NRXN1*. Both of these genes are large (>1 MB) and included over 100 SNPs from the genotyping array. In order to avoid false positives with our small sample size, we chose to focus our analysis on regions that have previously been associated with neurodevelopmental disorder, analysing all genotyped SNPs (after quality control steps described above (see “DNA collection and analysis”) within these selected regions.

In *CNTNAP2* ( NM_014141), we focused on a region spanning exons 13-14 (chr7:147,514,390-147,612,852 (hg19)). This region includes a cluster of 9 SNPs previously associated with language disorder ( Vernes *et al.*, 2008; Whitehouse *et al*., 2011). We had direct genotype data for 22 SNPs across this region. In addition, we used imputation to obtain genotypes for SNPs rs2710102 and rs7794745. These were the first SNPs reported to be associated with ASD, and represent the two main SNPs used in the majority of association studies in neurodevelopmental disorders ( Alarcón *et al*., 2008; Arking *et al*., 2008). These two SNPs were not directly genotyped on the Illumina arrays and were therefore imputed for all individuals. Imputation was performed on the Michigan Imputation Server, an online server which generates phased and imputed genotypes using high-density reference panels. Variant Call Files (VCF) were uploaded for 15,936 SNPs genotyped on chromosome 7. Genotypes were phased within Eagle and imputed by Minimac against the Human Reference Panel hrc.r1.1.2016, which includes 64,940 haplotypes of predominantly European ancestry. In total, genotypes were generated for 2,289,829 SNPs across chromosome 7,513,970 of which had quality scores 0.9. The two SNPs of interest, rs2710102 and rs7794745 had quality scores of 0.9938 and 0.94127 respectively.

The second candidate is *NRXN1* ( NM_004801). Although this gene met our criteria of being relevant for both synaptic function and neurodevelopmental phenotypes, the studies showing this link involved deletions rather than common variants ( Ching *et al*., 2010). A recent analysis of clinical microarray data showed that deletions in exons near the 5′ end of *NRXN1* were specifically implicated in neurodevelopmental disorders ( Lowther *et al*., 2017). Accordingly, we focused on 23 SNPs in this region of the gene chr2:51,141,501-51,280,121 (hg19). These SNPs covered exons 1–4 plus 20 Kb upstream (5') of the gene as this region includes important regulatory sequences. Details of the SNPs included in the analysis are shown with Appendix material.

The SNPs within the chosen regions were filtered for minor allele frequency and Hardy Weinberg Equilibrium (as outlined in DNA collection and analysis) but were not pruned for linkage disequilibrium. Previous simulations indicate that the GSCA method is not greatly affected by linkage disequilibrium (see Romdhani *et al.*, 2014). Across the *CNTNAP2* region, six pairwise combinations of SNPs had R2 > 0.8. Across the *NRXN1* region, 8 pairwise combinations of SNPs had R2 > 0.8. Details of the SNPs included in the analysis and a table of correlations between SNPs are shown with Appendix material.

### Statistical methods

**Figure 4.**
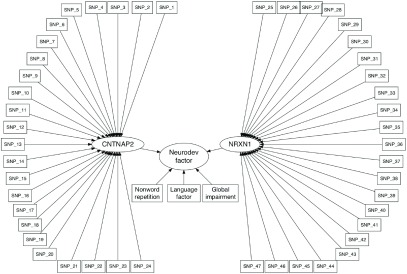
Structural equation diagram for analysis. The GSCA analysis estimates the path from each gene to the Neurodev factor, with significance calculated by permutation analysis.

*CNTNAP2* and *NRXN1* genes from the trisomy sample will be analysed for association with a latent variable based on the three phenotypes using a structural equation modelling (SEM) approach adapted for genetic analysis ( Romdhani *et al*., 2015). The model specification for our analysis is shown in Figure 4.

Romdhani *et al*. (2015) used the Generalized Structured Component Analysis (GSCA) developed by Hwang and Takane (2004). This method uses component-based path modelling rather than traditional covariance-based SEM, allowing adequate model fit to be achieved when using smaller samples ( Chin & Newstead, 1999; Tenenhaus *et al.*, 2005). The measurement models in the SEM framework are not typical regression format using latent factors; instead they are fitted using alternating least squares to estimate the weights and parameters, which is similar to principal components analysis. The advantage of this approach is that it does not attempt to fit the whole covariance matrix for observed and latent variables, but rather fits a separate measurement model for the contribution of observed variables to each latent factor, as well as a covariance model for the latent factors. Hence, we do not estimate the contribution of individual SNPs in each gene to the phenotype; rather, their influence is represented via the weighted sum. Similarly, the latent phenotypic factor (termed Neuro in Figure 4) is a weighted sum of the three measures of the phenotype. We will estimate the significance of one direct pathway from the *CNTNAP2* gene to the latent phenotype, and one from *NRXN1* to the latent phenotype. This method thus gives a single estimate of the overall impact of SNPs in a region of the phenotype.

We conducted simulations that indicated that this method is feasible with the number of SNPs and phenotypes in our sample (see Appendix 5): the permutation method, used by this approach to effectively quantify the test statistic distribution, generates p-values independently for each path, and a correction is required to take this into account. Because the evidence of association of common variants was stronger for *CNTNAP2* than for *NRXN1*, we used a sequential approach to setting a significance level (alpha), using a critical p-value of .05 to test the pathway from *CNTNAP2* to the Neuro factor, and .025 for the pathway from *NRXN1* to the Neuro factor.

In addition, we will do the same analyses with children from the two comparison samples.

We predict that one or both paths from *CNTNAP2* and *NRXN1* to the Neurodev factor will indicate significant association in the sex chromosome trisomy sample. We further predict that any associations in the comparison samples will be similar in direction, but smaller in size and may not reach statistical significance.

Any additional analyses of subgroups or phenotypes suggested by inspecting the data will be treated as exploratory and in need of replication in another sample.

## Self-certification statement

The authors confirm that they had no prior access to the full dataset proposed for analysis.

The neurodevelopmental data has already been processed by DB and PT to derive the phenotypes to be used in the analysis; DFN has processed the DNA data separately to decide on the genotypes. The key tests proposed in this protocol will involve putting the two strands of data together, which has deliberately not been done, so that predictions can be derived without being aware of the data.

## Supplementary material

Appendices 1–5 are available on Open Science Framework
