# Supplementary material for: Stage 1 Registered Report: Variation in neurodevelopmental outcomes in children with sex chromosome trisomies: protocol for a test of the double hit hypothesis
Source: Wellcome Open Res. 2018 Apr 24;3:10. Originally published 2018 Feb 12. [Version 2] doi: 10.12688/wellcomeopenres.13828.2 (PMC5904730; doi:10.12688/wellcomeopenres.13828.2)
Supplement: Peer review history

# Peer review: Referee response for version 2

**DOI:** 10.21956/wellcomeopenres.15855.r32984

**Published:** 2018-05-16

**Authors:** Armin Raznahan

**Affiliations:** 1 Developmental Neurogenomics Unit (DNU), National Institute of Mental Health (NIMH) , Bethesda, MD, USA

I have read this submission. I believe that I have an appropriate level of expertise to confirm that it is of an acceptable scientific standard.

# Peer review: Referee response for version 2

**DOI:** 10.21956/wellcomeopenres.15855.r33083

**Published:** 2018-05-04

**Authors:** David Skuse

**Affiliations:** 1 Behavioural & Brain Sciences Lab, UCL Great Ormond Street Institute of Child Health, London, UK

I have read this submission. I believe that I have an appropriate level of expertise to confirm that it is of an acceptable scientific standard.

# Peer review: Referee response for version 1

**DOI:** 10.21956/wellcomeopenres.15031.r31601

**Published:** 2018-04-06

**Authors:** David Skuse

**Affiliations:** 1 Behavioural & Brain Sciences Lab, UCL Great Ormond Street Institute of Child Health, London, UK

*Background:*

 The proposed analysis is predicated on a clinical observation that individuals with sex chromosome duplication aneuploidies (47,XXX; 47,XXY; 47,XYY) have an increased rate of social communication dysfunction, including language. Intra-aneuploidy variability in the severity of that phenotype remains unexplained. Newbury and colleagues hypothesize that functionally significant variants of certain autosomal genes (CNTNAP2 and NRXN1), which have a well-established role in neurodevelopment, could serve to amplify the detrimental impact of sex chromosome aneuploidy.

 The conceptual basis of this study can be found in Bishop and Scerif ^1^. The authors noted there are similarities between the phenotype of Klinefelter syndrome (XXY) and that of  individuals who suffer from a Specific Language Impairment. They suggested that neuroligin genes on the sex chromosomes (specifically NLGN3 and NLGN4) would be expressed in excessive dosage in XXY syndrome, with a potential impact on the risk of neurodevelopmental syndromes (NDS). They proposed an epistatic interaction occurs between NLGN3/4 and a specific autosomal gene on chromosome 7 (CNTNAP2), variants of which had been shown to associate with language problems in previous studies. Neither genetic risk factor (sex-linked or autosomal) would be sufficient to cause SLI alone; not all XXY males have language problems, and many CNTNAP2 risk variants are inherited from an apparently unaffected parent.  However, in combination (excessive NLGN3/4 expression plus risk variants of CNTNAP2), there could be some multiplicative impact of genotypic risk that resulted in a clinical (language-related) phenotype. 

 In their updated hypothesis, Bishop and her colleagues hypothesise that the sex-linked genetic risk factor in the aneuploidy is most likely to be NLGN4X. Not only is there an equivalent gene on the Y chromosome (NLGNY) but NLGN4X escapes X-inactivation (unlike NLGN3X), and therefore could be expressed in increased dosage in duplication syndromes such as XXY. Does NLGN4X/Y impact language development? A recent review of genomic influences on language development Graham and Fisher (2015 ^2^) do not mention NLGN4, but of course that does not negate the possibility that major expression changes due to aneuploidy could have influence outside the normal range of their activity.

*Neuroligins:*

 Neuroligins are post-synaptic transmembrane proteins and form complexes with neurexins (as shown in Figure 1). There are four neuroligins expressed in humans, NLGN1 (3q26.3), NLGN2 (17p13) on the autosomes and NLGN3 (Xq13.1) & NLGN 4 (Xp22.32) on the X chromosome ^3^. 

 NLGN4Y on the Y chromosome is almost identical structurally to NLGN4X ^4^. Neuroligins NLGN4X and NLGN4Y are both expressed in the brain. Whilst there is no specific study on the influence of X-chromosome duplication on risk of ASD, there is evidence that NLGN4Y overexpression has a role in the pathogenesis of ASD in XYY syndrome ^5^. 

 Note that there could be a difference in X and Y-linked neuroligins’ role in neurodevelopmental processes, both in terms of where in the brain the genes are expressed and in the degree to which they are expressed, despite the fact that the two structural variants of the NLGN4 gene are very similar to one another. 

*Neurexins:*

 Neurexins are in the same functional network as neuroligins, and form complexes with them at neuronal synapses. There are three NRXN genes, NRXN1, NRXN2 and NRXN3. Their trans-synaptic cell-adhesion role mediates essential signaling between presynaptic and postsynaptic domains. Mutations in neurexins have been widely implicated in cognitive impairment and predisposition to psychiatric disorders.

 CNTNAP2 encodes a neurexin-like cell adhesion molecule (Caspr2) but the impact of specific CNTNAP2 genetic variants (and the functional integrity of Caspr2) on brain development is still not well understood ^6^. Interestingly, the expression of CNTNAP2 is regulated by the FOXP2 transcription factor (itself linked to speech/language disorders). Whilst the neuroligin-neurexin transmembrane complex has substantial supporting evidence, Figure 1 implies there is an equivalent transmembrane connection between ‘CNTNAPs’ and Contactins/Catenins, but the evidence for that is weak ^7^. A recent review indicated the main role of contactin-associated proteins (CNTNAPs) is outside the synapse, in neuron–glia interactions in myelinated axons ^8^. 

*Experimental procedure:*

 The experimental procedure proposed is to examine genetic risk for a disordered language phenotype in an already collected unbiased sample of X-chromosome aneuploidies, encompassing girls with XXX trisomy, and boys with XXY and XYY syndromes. A comparison sample of twins participating in a study of language and laterality has been chosen (two separate samples of 184/186 children). The phenotypic assessment battery is comprehensive and sensibly treats phenotypes as quantitative on the whole (although the classification from the DAWBA psychiatric phenotypes is nominal).

*Cognitive deficits and ASD risk in sex chromosome aneuploidies:*

 A recent review of sex-chromosome aneuploidies and their cognitive consequences ^9^ points out that XXY and XYY boys tend to have lower verbal than nonverbal IQ. In XXX syndrome both nonverbal and verbal abilities tend to be equally impaired, and verbal skills are equivalent to those of XXY males. In fact, in all three aneuploidies the degree of verbal impairment is similar (0.7-1.0 SDS), which implies a common mechanism may be influencing language development. It is worth noting, however, that in 45,X (X0-Turner) syndrome there is no significant verbal IQ/speech/language deficit; it seems duplication of a sex-chromosome risk factor is more detrimental than haploinsufficiency in respect of speech/language deficits. 

 Both XXY and XYY syndromes are associated with a high risk of ASD, though not XXX. There is a substantially increased prevalence of ASD in association with X0 (up to 30%), which indicates a dissociation between the processes underlying ASD risk and language impairment in sex chromosome aneuploidy. To be specific, XXX females have relatively low risk of ASD despite language impairments that are equivalent in severity to XXX and XXY (where ASD risk is high): X0 females have a high risk of ASD but they do not typically experience any significant language impairments. There is a possible confounding impact of hormonal variables: XXX females are fertile, X0 females are not. Many XXY males are androgen deficient, XYY males are not ^10^. 

*Role of the Y-chromosome and partial X-inactivation:*

 Newbury and colleagues report that in their sample the proportion of XYY males with normal-range cognition was just 14% but in XXX syndrome was four times greater (55%). The figure for XXY males was intermediate (37%). Speech and language problems were also more common along the same gradient ^1^, 24% in XXX, 47% in XXY and 71% in XYY. 

 What could account for that gradation of risk? 

 First, NLGN4Y may have a different expression pattern in the brain from NLGNX ^11^ and therefore over-expression may be more detrimental to key regions of the ‘social brain’. There is emerging evidence that Y-linked genes are subject to increased expression in duplication syndromes ^5^. Hence, the effect of having an additional Y chromosome may be particularly impairing, and different in its impact on brain development to an additional X-chromosome.

 Second, there is evidence that although there is homology between the PAR1 genes in males and females (i.e. between X and Y allelic variants), in general pseudoautosomal genes in the PAR1 region of males are expressed at a higher level than those in females ^12^^-^^13^ implying that aneuploidy for the Y chromosome could be more detrimental than aneuploidy for the X-chromosome.

 Third, although a key element of the Bishop et al hypothesis is that NLGN4X escapes X-inactivation (XCI) and therefore will be over-expressed in XXX females and XXY males, the gene lies outside PAR1 and its escape from inactivation is only partial (average 26%). This partial expression is substantially lower than most X-linked genes that escape inactivation. More significantly, in respect to its implications for the Bishop/Scerif hypothesis, expression of NLGN4X from the ‘inactive’ X- chromosome is highly variable between typical females. The proportion of females in which escape of this gene from XCI has been recorded is only 38% ^12^^-^^13^. However, we must acknowledge that the situation in aneuploidies has not been studied.

*An alternative hypothesis:*

 Accordingly, without the need to evoke an epistatic relationship between NLGN4X and Y and neurexin-associated SNVs, there is good evidence that within-karyotype variation could be explained on the basis of variability in expression of genes from the sex chromosomes, particularly within the female sample with XXX, and XXY males. On the basis of this alternative hypothesis, we would expect to find the least variability in language-related phenotype in the XYY male sample, and arguably the most variable phenotypes in the XXX sample. These data are already available to Newbury et al, hence the alternative hypothesis could easily be tested.

*The role of CNTNAP2 and NRXN1 SNPs:*

 Coming onto the proposed interaction between the NLGNs and genotypic variants of NRXN1 and CNTNAP2, what is the evidence that the SNPs under investigation are themselves risk factors for language problems (with or without associated ASD)? Two particular SNPs of interest, according to the proposal, are rs2710102 and rs7794745 (both of which are in introns); they have been extensively researched in association studies of neurodevelopmental disorder. 

 Support for the Bishop et al hypothesis would derive from a finding that ‘risk variants’ of the autosomal genes CNTNAP2 and NRXN1 should have some phenotypic impact on brain structure/function in neurotypical populations; compelling evidence could be adduced from neuroimaging, comparing brain structure/function between populations with the variants of interest.

 Uddén et al (2017 ^14^) attempted to confirm findings made by earlier neuroimaging studies that claimed the rs7794745 variant of CNTNAP2 impacted grey matter in regions of the ‘social’ brain plausibly associated with ASD. Using a substantially larger sample of typical subjects they found that the risk variant associated with rs7794745 did link significantly to grey matter changes in the visual dorsal stream area of the occipital lobe. However, they could not find evidence of changes to volume changes in regions more strongly implicated in autistic traits (such as frontal or temporal areas). They express caution about potential misinterpretation of neuroimaging findings when small sample studies are performed. 

 The evidence supporting the other main CNTNAP2 variant of interest to Newbury et al, rs2710102, is not strong on the basis of evidence from typical populations. In a replication study, Dennis et al (2011 ^15^) concluded that it may be a polymorphism that, when combined with others, could increase the risk for autism by enhancing the susceptibility to language disorders but there are countervailing findings too ^16^.

 The relevance of NRXN1 to the hypothesis comes, as Bishop et al acknowledge, from evidence that exonic deletions at the 5’ end of the gene are associated with a substantially increased risk of neurodevelopmental dysfunction in terms of cognition and psychiatric risk ^17^. In the proposed study the region will be reasonably well covered with 23 SNPs. 

 On the other hand, if polymorphic variation in this gene were associated with neurodevelopmental problems in a non-clinical population (as opposed to deletion of exons in clinical cases) there should be some published evidence. Wang et al, (2018 ^18^) recently found no association between two SNPs in NRXN1 and a clinical phenotype among a Chinese mixed population of ASD and controls (although neither of those SNPs is listed in Appendix 7 of the proposal). Where is the evidence that, in a typical population, SNP variants at the 5’ end of NRXN1 are associated with language development?  

*Statistical analysis:*

 Finally, there are novel statistical methods to be used for the analysis of this sample, which have been devised to get around the problem of limited power (due to small sample size). The Romdhani et al analysis claims to permit adequate model fit using smaller samples than conventional methods of association analysis. A weighted influence of a sum of SNPs is represented (Figure 4), implying the impact of a concatenation of variants within the critical region of candidate genes can be treated as a ‘weighted score’, irrespective of the contribution of individuals SNPs in each gene to the phenotype. This seems highly speculative, whereas the application of similar techniques to the phenotypic measures is uncontroversial. The Romdhani method’s application to multiple SNPs in a relatively small sample has, so far as I can ascertain, not been applied in other studies to date.

*Summary:*

 Bishop and her colleagues are to be congratulated for the work they have done in assembling a relatively large sample of X-chromosome aneuploidies and for the careful phenotyping they have conducted to date. 

 Copy number variants (CNV), both duplications and deletions, can be detrimental to neurodevelopment and some (such as those affecting the 16p11.2 region) can impact on language development ^19^. It would have been worth checking the samples of aneuploidy collected by this team with a microarray for possible ‘second hits’ ^20^. It is also the case that many potentially pathogenic autosomal CNV have variable expression phenotypically and may be inherited from an apparently unaffected parent. Polygenic background variation (as well as environmental factors) are often cited as a modifying factor, accounting for the variable penetrance ^21^. 

 In summary, it is frequently the case that inappropriate gene dosage syndromes involving duplication or deletion of critical regions of the genome to have variable phenotypic consequences on neurodevelopment between affected individuals. The proposal from Newbury et al aims to test a specific hypothesis, which entails making the assumption that the risk of a particular phenotype (detriment to language) is raised to a *consistent*(subclinical) threshold by X- or Y chromosome duplication. The biological mechanism is assumed to be NLGN4X/Y overexpression. The impact on an affected individual’s observable phenotype is, they propose, further modified by functional variants (SNPs) on the autosomal genes CNTNAP2 and NRXN1. It is these SNPs that contribute a range of variable risk (risk that contributes to the polygenic background of the general population) that could, in certain specific combinations, tip the balance toward a phenotype characterized by a clinically significant disorder of language. 

 The proposal that inter-aneuploidy variation on the sex chromosomes could be strongly influenced by NLGN4X/Y expression is a plausible one. There are novel elements to the Bishop et al hypothesis that are worth testing, in particular the possibility that Romdhani’s approach to measuring aggregated SNP risk in small samples is valid. However, on the basis of recently discovered evidence concerning the characteristics of homologous X- and Y-linked genes, it seems to me that intra-aneuploidy modifying factors on language and social communication skills are more likely to be associated with the characteristics of variable sex chromosome gene expression than with SNPs in the two autosomal candidates, CNPNAP2 and NRXN1.

**Have the authors pre-specified sufficient outcome-neutral tests for ensuring that the results obtained can test the stated hypotheses, including positive controls and quality checks?**

 Partly.

I have read this submission. I believe that I have an appropriate level of expertise to confirm that it is of an acceptable scientific standard, however I have significant reservations, as outlined above.

## References

[1] BishopDVScerifG: Klinefelter syndrome as a window on the aetiology of language and communication impairments in children: the neuroligin-neurexin hypothesis. .2011;100(6) : 10.1111/j.1651-2227.2011.02150.x 903-7 10.1111/j.1651-2227.2011.02150.x 21418292PMC3107947

[2] GrahamSAFisherSE: Understanding Language from a Genomic Perspective. .2015;49: 10.1146/annurev-genet-120213-092236 131-60 10.1146/annurev-genet-120213-092236 26442845

[3] SüdhofTC: Neuroligins and neurexins link synaptic function to cognitive disease. .2008;455(7215) : 10.1038/nature07456 903-11 10.1038/nature07456 18923512PMC2673233

[4] BembenMAShipmanSLNicollRARocheKW: The cellular and molecular landscape of neuroligins. .2015;38(8) : 10.1016/j.tins.2015.06.004 496-505 10.1016/j.tins.2015.06.004 26209464PMC9381026

[5] RossJLTartagliaNMerryDEDalvaMZinnAR: Behavioral phenotypes in males with XYY and possible role of increased NLGN4Y expression in autism features. .2015;14(2) : 10.1111/gbb.12200 137-44 10.1111/gbb.12200 25558953PMC4756915

[6] CanaliGGarciaMHivertBPinatelDGoullancourtAOguievetskaiaKSaint-MartinMGiraultJFaivre-SarrailhCGoutebrozeL: Genetic variants in autism-related CNTNAP2 impair axonal growth of cortical neurons. .2018; 10.1093/hmg/ddy102 10.1093/hmg/ddy102 29788201

[7] GilbertJManHY: Fundamental Elements in Autism: From Neurogenesis and Neurite Growth to Synaptic Plasticity. .2017;11: 10.3389/fncel.2017.00359 359 10.3389/fncel.2017.00359 29209173PMC5701944

[8] PeñagarikanoO: CNTNAP2 Mutations in Autism.2016; 10.1016/B978-0-12-800109-7.00012-1 177-188 10.1016/B978-0-12-800109-7.00012-1

[9] PrintzlauFWolstencroftJSkuseD: Cognitive, behavioral, and neural consequences of sex chromosome aneuploidy. .2017;95(1-2) : 10.1002/jnr.23951 311-319 10.1002/jnr.23951 27870409

[10] SCHIAVIROWENDFOGELMWHITEDSZECHTERR: PITUITARY-GONADAL FUNCTION IN XYY AND XXY MEN IDENTIFIED IN A POPULATION SURVEY. .1978;9(3) : 10.1111/j.1365-2265.1978.tb02205.x 233-239 10.1111/j.1365-2265.1978.tb02205.x 709893

[11] JohanssonMMLundinEQianXMirzazadehMHalvardsonJDarjEFeukLNilssonMJazinE: Spatial sexual dimorphism of X and Y homolog gene expression in the human central nervous system during early male development. .2016;7: 10.1186/s13293-015-0056-4 5 10.1186/s13293-015-0056-4 26759715PMC4710049

[12] TukiainenTVillaniACet.al.: Landscape of X chromosome inactivation across human tissues. .2017;550(7675) :244-248 10.1038/nature24265PMC568519229022598

[13] CarrelLBrownCJ: When the Lyon(ized chromosome) roars: ongoing expression from an inactive X chromosome. .2017;372(1733) : 10.1098/rstb.2016.0355 10.1098/rstb.2016.0355 28947654PMC5627157

[14] UddénJSnijdersTMFisherSEHagoortP: A common variant of the CNTNAP2 gene is associated with structural variation in the left superior occipital gyrus. .172: 10.1016/j.bandl.2016.02.003 16-21 10.1016/j.bandl.2016.02.003 27059522

[15] DennisELJahanshadNRudieJDBrownJAJohnsonKMcMahonKLde ZubicarayGIMontgomeryGMartinNGWrightMJBookheimerSYDaprettoMTogaAWThompsonPM: Altered structural brain connectivity in healthy carriers of the autism risk gene, CNTNAP2. .2011;1(6) : 10.1089/brain.2011.0064 447-59 10.1089/brain.2011.0064 22500773PMC3420970

[16] WerlingAMBobrowskiETaurinesRGundelfingerRRomanosMGrünblattEWalitzaS: CNTNAP2 gene in high functioning autism: no association according to family and meta-analysis approaches. .2016;123(3) : 10.1007/s00702-015-1458-5 353-63 10.1007/s00702-015-1458-5 26559825

[17] LowtherCSpeevakMArmourCMGohESGrahamGELiCZeesmanSNowaczykMJSchultzLAMorraANicolsonRBikangagaPSamdupDZaazouMBoydKJungJHSiuVRajguruMGoobieSTarnopolskyMAPrasadCDickPTHussainASWalingaMReijengaRGGazzelloneMLionelACMarshallCRSchererSWStavropoulosDJMcCreadyEBassettAS: Molecular characterization of NRXN1 deletions from 19,263 clinical microarray cases identifies exons important for neurodevelopmental disease expression. .19(1) : 10.1038/gim.2016.54 53-61 10.1038/gim.2016.54 27195815PMC4980119

[18] WangJGongJLiLChenYLiuLGuHLuoXHouFZhangJSongR: Neurexin gene family variants as risk factors for autism spectrum disorder. .2018;11(1) : 10.1002/aur.1881 37-43 10.1002/aur.1881 29045040

[19] BlackmonKThesenTGreenSBen-AviEWangXFuchsBKuznieckyRDevinskyO: Focal Cortical Anomalies and Language Impairment in 16p11.2 Deletion and Duplication Syndrome. .2017; 10.1093/cercor/bhx143 1-9 10.1093/cercor/bhx143 28591836

[20] GirirajanSRosenfeldJACoeBPParikhSFriedmanNGoldsteinAFilipinkRAMcConnellJSAngleBMeschinoWSNezaratiMMAsamoahAJacksonKEGowansGCMartinJACarmanyEPStocktonDWSchnurREPenneyLSMartinDMRaskinSLeppigKThieseHSmithRAbergENiyazovDMEscobarLFEl-KhechenDJohnsonKDLebelRRSiefkasKBallSShurNMcGuireMBrasingtonCKSpenceJEMartinLSClericuzioCBallifBCShafferLGEichlerEE: Phenotypic heterogeneity of genomic disorders and rare copy-number variants. .2012;367(14) : 10.1056/NEJMoa1200395 1321-31 10.1056/NEJMoa1200395 22970919PMC3494411

[21] RosenfeldJACoeBPEichlerEECuckleHShafferLG: Estimates of penetrance for recurrent pathogenic copy-number variations. .2013;15(6) : 10.1038/gim.2012.164 478-81 10.1038/gim.2012.164 23258348PMC3664238

# Response

**Published:** 2018-04-16

**Authors:** Dorothy Bishop

**Affiliations:** University of Oxford, UK

This review gave an excellent overview of background material relevant to our study. Here we respond just to comments that raised questions about our planned approach.

 P 13, para 1. The Graham and Fisher review provides a masterly overview of associations with language-related disorders found in GWAS, and we now add a mention of this. For two reasons, we adopted a different approach to those authors to identify candidates for analysis: first, we had a specific hypothesis that led us to focus on genes involved in synaptic function, and second, we were conscious that if we analysed a very large number of candidate SNPs, we would compromise our analysis, given that the small sample limited our statistical power. Accordingly, we took a systematic approach to homing in on candidates, searching for genes that met the dual criteria of being mentioned in association with language-related phenotypes, and being involved in synaptic function. This threw up some genes that have not traditionally been regarded as involved in language function because our search term picked up studies that included language phenotypes beyond SLI or dyslexia: notably autism or schizophrenia, where language is affected. These seemed worth exploring, because, as noted by Graham and Fisher, the boundaries between neurodevelopmental disorders are fuzzy, especially in the context of sex chromosome trisomies (SCTs), where a wide range of phenotypes can be seen.

 P 14, para 4. We have modified our language to indicate that much about how CNTNAP2 operates is still not understood. Nevertheless, we argue that there is supportive evidence for a role of CNTNAP2 in synaptic development. See, for instance, Lu, Z., Reddy, M. V. V. V. S., Liu, J., Kalichava, A., Liu, J., Zhang, L., . . . Rudenko, G. (2016). Molecular architecture of Contactin-associated Protein-like 2 (CNTNAP2) and its interaction with Contactin 2 (CNTN2). Journal of Biological Chemistry, 291(46), 24133-24147. doi:10.1074/jbc.M116.748236

 P 14, para 6-7. We would query the idea that there is such a clear division between risk for language impairment and risk for ASD in different trisomies. The study cited by Printzlau et al cites Bishop et al as finding no increase in risk of ASD in girls with XXX. That was so, but the ASD rates were based solely on parental report of a diagnosis, and it is increasingly recognised that girls are underdiagnosed. Furthermore, in that same study, parental report on CCC-2, which assesses autistic-like language features, the girls with XXX showed evidence of impairment in pragmatic skills, scoring well below the unaffected sibling group. We are in the process of writing up a detailed account of the phenotypic data that we have on our current sample; one conclusion that we will draw is that variation within a karyotype is considerably greater than variation between karyotypes. Overall, our data are consistent with the idea that all three karyotypes create an increased risk for language difficulties and ASD symptoms. The fact that language is typically unaffected in X0 girls, despite increased risk for ASD, is indeed interesting in suggesting that the impact of deletion differs from that of duplication. This is a point we think relevant for a later Discussion section.

 P 15, para 2. The estimates of proportion of cases with normal-range cognition do not come from Newbury et al – they do correspond to figures reported by Bishop & Scerif that relate to the Bishop et al (2011) sample. However, the figures do *not* refer to ‘normal range cognition’ but rather to cases where there was no neurodevelopmental or educational diagnosis, from parental report. Many of the problems that were reported concerned behaviour rather than cognition, notably ADHD and ASD. For data on cognitive function, a better source is the systematic review by Leggett et al (2010), which focused on neonatally identified samples and found verbal ability impaired in all three karyotypes, but lower nonverbal IQ in the XXX females.  So while we agree that there is potential interest in comparing the three karyotypes as they could potentially throw light on underlying mechanisms, we think it is justifiable to treat them together for this specific analysis to ascertain whether genetic background can explain phenotypic variation in language phenotypes.

 P 15, paras 3-4. These potential explanations for greater impact of NLGN4Y compared to NLGN4X are plausible and interesting. If the genetic background from candidate genes exerts any impact, we can consider whether any further variance is explained by karyotype (see Analysis Plan, Subgroups), but note we are underpowered to detect karyotype differences unless they are very large, and the ascertainment bias in our sample means that we cannot meaningfully interpret main effects on phenotype (see end para 1, participants).

 P 15, para 4. We included genes from PAR1 in our literature search and found none was associated with language phenotypes.

 P 15, para 5-6. We agree that variation in escape from inactivation for NLGN4X could explain phenotypic variation; we are not in a position to assess variable gene expression, but as Skuse notes, if this mechanism is important, then we should see greater phenotypic heterogeneity in karyotypes with an extra X than in the XYY group. We can indeed report those results, but we again would note that interpretation would have to be cautious, given the ascertainment bias. A higher percentage of boys with XYY had the trisomy identified because they were having problems (see Figure 2, p 5); if we restrict consideration only to the unbiased group, the numbers are very small. We have now mentioned this alternative hypothesis in the text, but also pointed out that testing it with our data carries a high risk of type II error.

 P 15-16. SNPs as risk factors

 The exact definition of “risk variant” is a very hard thing! As Skuse points out, even when particular SNPs are robustly implicated, we often see flipping between risk alleles. Our approach departs from that usually adopted, because we are not convinced that a focus on specific SNPs will be illuminating. We therefore have chosen to perform an analysis of all SNPs across risk regions capturing maximum information across these regions rather than relying on specific SNPs which may well be proxies for functional variants.

 P 16, para 3 and 4. We agree with the reviewer that prior literature on SNPs associated with language has not always led to convincing results and strong claims have sometimes been based on woefully underpowered studies. Our review of the neurogenetics field (Grabitz et al , 2017) is one reason why we decided we should preregister our proposed analysis, as it is just too easy to find something in a dataset if you have enough potential genotypes and phenotypes and a small sample.

 P 16, para 5. We agree that the predicted association of NRXN1 with language phenotypes is a long shot; the reviewer is correct – we are aware of no prior reports of association with common variants. This is why this is our second hypothesis. However, while the evidence for language association is far less convincing than for CNTNAP2, in terms of its role in neural circuits, NRXN1 is a strong candidate. We noted in the text that there was evidence that deletions near the 5’ end of *NRXN1* were specifically implicated in neurodevelopmental disorders ( Lowther *et al*., 2017). Potentially, the SNP analysis will tag variations and deletions in this region: if a SNP falls within a region of deletion then we would expect to see a loss of heterozygosity across that region which will alter the allele frequencies of the SNPs accordingly. Furthermore, surrounding SNPs may be in LD with the presence of deletions. To be honest, we’ll be surprised if we find anything with NRXN1, but it emerged from our literature search and we think it’s worth looking at.

 P 16, para 7: comment on the analysis. We agree that our approach to analysis has not been applied to other studies in this area, and this is a high risk approach. We decided to adopt this method because it appeared to be the best way of optimising power in an analysis with such a small sample. If we do find that it reveals results of interest, it would suggest it might be worth using this method more widely: we do think the traditional approach of looking at many SNPs and correcting for the number of SNPs may obscure genuine associations.

 P 17. Para 1: We will be looking at CNVs in a separate analysis; this is a natural extension to the work of Newbury and Simpson, who have already looked at samples with SLI. Although CNVs can be looked at in the context of a double hit model, this is complicated by an alternative possibility, that those with a trisomy may also carry an additional burden of CNVs. We feel therefore that this merits a separate report.

# Peer review: Referee response for version 1

**DOI:** 10.21956/wellcomeopenres.15031.r31717

**Published:** 2018-03-26

**Authors:** Beate St Pourcain

**Affiliations:** 1 Language and Genetics Department, Max Planck Institute for Psycholinguistics, Nijmegen, Netherlands

Newbury and colleagues submitted a Wellcome Open Research Stage 1 registered report studying neurodevelopmental outcomes in children with sex chromosome trisomies (SCTs), who show an increased rate of neurodevelopmental difficulties involving language. Adopting a double-hit hypothesis, the authors assume that the presence of an additional sex chromosome will amplify the impact of common autosomal genetic variants residing within genes involved in synaptic functionality. The authors specifically focus on the functional network of neuroligins involving CNTNAPs and Neurexins, as encoded by the selected candidate genes *CNTNAP2 and NRXN1* and will study common variation at these loci.

 I have the following comments:

1. XXX females and XXY males (Klinefelter's syndrome) show X chromosome inactivation, while XYY males do not, suggesting additional mechanisms that may affect gene dosage. Also there seems to be a possibility for differences among SCTs groups with respect to the proportion of children affected by a history of speech and language-therapy (24% for XXX girls vs 71% for XYY boys). Thus, the statement that common mechanism will apply regardless of karyotype may not be as unequivocally applicable as assumed. Could the authors clarify their statements on karyotype? Why could individuals with karyotypes involving X chromosome inactivation not be lumped together as part of a sensitivity analysis? Also, the authors may consider whether the severity of the outcome depends on common variation that influences (random) X chromosome inactivation e.g. within *XIST* and *TSIX* genes.
2. The authors assume that risk to developmental language disorder (DLDs) as carried by common variation, observed in individuals with a normal karyotype, is exacerbated in the context of SCTs. This is a novel and very provocative hypothesis that can provide deeper insight into the aetiology of DLDs. However, it is also known that common variation contributing to risk of developing ASD in children with and without *de novo* structural variation might act through slightly different pathways (Weiner et al., 2017 ^1^). This suggests that also common variation acting on the background of chromosomal abnormalities may invoke partially different aetiological mechanisms. If possible, within the realm of adequate power, I would therefore recommend extending the scope of common variation tested. Nonetheless, the hypotheses proposed in this report are biologically plausible and, more importantly, testable with adequate power and will provide an improved understanding of the genetic mechanisms contributing to DLD. 
3. The statistical analysis using partial least squares path modelling is sophisticated and adequate to detect joint common variant effects. The description of variants selected could be slightly improved, however, especially with respect to handling LD patterns.

In conclusion, this report proposes a very interesting and valuable study that can provide insight into the aetiological mechanisms underlying DLDs using a sophisticated and thoughtful analysis approach studying a DLD high-risk population of children with SCTs. Investigating established candidate genes has furthermore implications for the understanding of the phenotype in the wider population of children with DLDs without karyotypic abnormalities.

I have read this submission. I believe that I have an appropriate level of expertise to confirm that it is of an acceptable scientific standard.

## References

[1] WeinerDJWigdorEMRipkeSWaltersRKKosmickiJAGroveJSamochaKEGoldsteinJIOkbayABybjerg-GrauholmJWergeTHougaardDMTaylorJiPSYCH-Broad Autism GroupPsychiatric Genomics Consortium Autism GroupSkuseDDevlinBAnneyRSandersSJBishopSMortensenPBBørglumADSmithGDDalyMJRobinsonEB: Polygenic transmission disequilibrium confirms that common and rare variation act additively to create risk for autism spectrum disorders. .2017;49(7) : 10.1038/ng.3863 978-985 10.1038/ng.3863 28504703PMC5552240

# Response

**Published:** 2018-04-16

**Authors:** Dorothy Bishop

**Affiliations:** University of Oxford, UK

Many thanks for the thoughtful comments on our protocol. 

1. The point about possible variation between karyotypes agrees with one of the main points raised by Skuse (see above). It makes sense that there are some variations across karyotypes that will need another explanatory account. Nevertheless, we think it worth starting by testing a general mechanism, for two reasons. First, language impairment seems to be more like height – just as there is a tendency to be tall in all three karyotypes, so there is a tendency to have poor language skills.  On our quantitative measures of language, the three groups do not look very different (though there is large variance around means). So there appears to be a general influence affecting all three karyotypes, even if there are karyotype-specific effects superimposed. NLGN4X does, at least in part, escape inactivation, so provides a plausible mechanism. Second, we have limited statistical power. If we take seriously correction for multiple comparisons, then we can either investigate several potential explanations with a stringent alpha, or reduce the number of tests we do and have a less stringent alpha. We have opted for the latter approach.
2. As noted in the text, focusing on two genes so we can optimise power  will not preclude us from doing further exploratory analyses, which would include investigating karyotype-specific effects, but we would then need to replicate findings in another sample. It would, of course, be great to extend the analyses and not just confine it to two candidates, but we would rapidly lose power if we applied appropriate corrections. More minor point: inclusion of variants on XIST and TSIX would pose problems as they are on the X chromosome.
3. Improved account of LD patterns – please see response to Raznahan below.

# Peer review: Referee response for version 1

**DOI:** 10.21956/wellcomeopenres.15031.r30762

**Published:** 2018-03-02

**Authors:** Armin Raznahan

**Affiliations:** 1 Developmental Neurogenomics Unit (DNU), National Institute of Mental Health (NIMH) , Bethesda, MD, USA

This stage 1 registered report describes a planned genetic association study relating common SNPs within two candidate genes to language and global neurodevelopment phenotypes in sex chromosome trisomy (SCT). 

 The motivating hypothesis for this study is that shared developmental difficulties across SCT could be due to altered dosage of the X-Y gametolog pair NLGN4X and NLGN4Y, but that variability in the severity of developmental difficulties may be explained by “double hits” in the form of common variation within autosomal genes implicated in synaptic mechanisms or language difficulties - CNTNAP2 and NRXN1.

 The specific hypothesis to be tested, by structural equation modeling of gene-behavior relationships, is that the SCT group will show stronger gene-behavior associations than euploidic controls. This hypothesis is based on the idea that gene dosage abnormality of Neuroligins will multiply the effects of functional variation in neurexins (CNTNAP2 and NRXN1) on language outcomes.

 The ideas motivating this report are biologically plausible, although the cited examples of epistasis do not involve copy number variations. The effects of gene dosage variation on gene function, and on the potential for interaction between functional variations in other genes are not necessarily the same as the effects of SNPs or SNVs.

 The proposed methods are suitable for testing the specific hypotheses to be tested. The authors take a thorough approach to the challenging issues to ascertainment bias in studies of SCT - adjusting their methods for power analysis and participant selection. The comparison group is twins rather than singletons, but the authors give reasons for why this might not bias their analyses. The behavioral/developmental phenotypes to be used are fit for purpose. The authors use transparent and systematic criteria for selecting their two autosomal genes of interest, but I could not locate details for their selection of regions within the genes besides the statement that they “focused their analysis on regions that have previously been associated with neurodevelopment disorder”. The genome addresses provided suggested that they are using linkage peaks perhaps, or CNV boundaries? It might be good to clarify this. Also once the regions are set, was linkage disequilibrium taken into account as part of selecting SNPS from target regions? Were SNPs entered into the SEM as linear ordinal variables, or were more complex SNP effects considered? It would be helpful to clarify these issues.

 In summary, notwithstanding the suggested additions of information above, this report describes a sound test of a plausible hypothesis for an important observation - marked inter-individual variability in phenotype severity within SCT. Addressing this question in SCT would have implications for the more general phenomenon of phenotypic variability in copy number variation syndromes.

**Have the authors pre-specified sufficient outcome-neutral tests for ensuring that the results obtained can test the stated hypotheses, including positive controls and quality checks?**

 Partly.

I have read this submission. I believe that I have an appropriate level of expertise to confirm that it is of an acceptable scientific standard, however I have significant reservations, as outlined above.

# Response

**Published:** 2018-04-16

**Authors:** Dorothy Bishop

**Affiliations:** University of Oxford, UK

Thank you for your encouraging comments. Here are our responses to specific points

P 21, para 5. Effects of gene dosage variation may not be same as SNPs or SNVs.

This is a fair point, though previous reports have implicated the double-hit hypothesis in relation to copy number variants encompassing other genes (for example, Newbury et al 2013 (PMID: 22909776), Girirajan et al 2009 (PMID: 20154674), and we now cite this work. In addition to the evidence from SNVs and biological plausibility, we feel this model is worth exploring in sex chromosome trisomies.

P 21, para 5 – need for more details for selection of regions

The regions selected were based upon previous association analyses of CNTNAP2 and NRXN1 in relation to language disorder. We have now updated the “Prioritising genotypes for analysis” section to make this clearer.

P 21, linkage disequilibrium taken into account?

We have now added this to the text:

“The SNPs within the chosen regions were filtered for minor allele frequency and Hardy Weinberg Equilibrium (as outlined in DNA collection and analysis) but were not pruned for linkage disequilibrium. Previous simulations indicate that the GCSA method is not greatly affected by linkage disequilibrium (see Romdhani et al 2014). A table of correlations between SNPs can be found in the Appendix. Across the CNTNAP2 region, six pairwise combinations of SNPs had R2 > 0.8. Across the NRXN1 region, 8 pairwise combinations of SNPs had R2 > 0.8.”
